# Minor Changes for a Major Impact: A Review of Epigenetic Modifications in Cell-Based Therapies for Stroke

**DOI:** 10.3390/ijms232113106

**Published:** 2022-10-28

**Authors:** Molly Monsour, Jonah Gordon, Gavin Lockard, Adam Alayli, Bassel Elsayed, Jacob Connolly, Cesar V. Borlongan

**Affiliations:** 1 University of South Florida Morsani College of Medicine, Tampa, FL 33602, USA; 2 Center of Excellence for Aging and Brain Repair, Department of Neurosurgery and Brain Repair, University of South Florida Morsani College of Medicine, Tampa, FL 33612, USA

**Keywords:** stroke, epigenetics, stem cells, neuroinflammation, atherosclerosis

## Abstract

Epigenetic changes in stroke may revolutionize cell-based therapies aimed at reducing ischemic stroke risk and damage. Epigenetic changes are a novel therapeutic target due to their specificity and potential for reversal. Possible targets for epigenetic modification include DNA methylation and demethylation, post-translational histone modification, and the actions of non-coding RNAs such as microRNAs. Many of these epigenetic modifications have been reported to modulate atherosclerosis development and progression, ultimately contributing to stroke pathogenesis. Furthermore, epigenetics may play a major role in inflammatory responses following stroke. Stem cells for stroke have demonstrated safety in clinical trials for stroke and show therapeutic benefit in pre-clinical studies. The efficacy of these cell-based interventions may be amplified with adjunctive epigenetic modifications. This review advances the role of epigenetics in atherosclerosis and inflammation in the context of stroke, followed by a discussion on current stem cell studies modulating epigenetics to ameliorate stroke damage.

## 1. Introduction to Stroke

Once termed the “incoming epidemic of the 21st century” by the World Health Organization, stroke is a major worldwide health concern and the 5th leading cause of death in the United States [1,2]. A complex multifactorial disease, the two main types of stroke include ischemic stroke and hemorrhagic stroke. Ischemic stroke accounts for around 78 percent of cases while hemorrhagic stroke accounts for the remaining 22 percent [3]. Available treatments for stroke are limited to tissue plasminogen activator (tPA) and mechanical thrombectomy to target clots [4]. While these acute treatments may be effective, they often have a window of only a few hours from the stroke event onset and are therefore not always provided to patients [4]. Studies of stroke patients have shown that the utilization rates of these two treatments are 15.7 percent for tPA and 5.4 percent for mechanical thrombectomy [5]. Even though these treatments have helped to reduce the overall death rate from stroke, future development of new treatments that promote plasticity within surviving brain tissue is the key to help lessen the burden of long-term disabilities [4].

The field of epigenetics has recently expanded to include the development of new treatments to target a variety of multifactorial diseases such as stroke. Epigenetic changes have emerged as a potential area for research because epigenetic aberrations are pharmaceutically reversible [6]. Possible targets for epigenetic modification include DNA methylation and demethylation, post-translational histone modification, and the actions of non-coding RNAs such as microRNAs (miRNAs) [6]. A family of DNA methyltransferases (DNMTs) catalyze DNA methylation, each with their own biologic function. DNMT3a and DNMT3b serve as the de novo DNMTs as they establish a new methylation pattern for previously unmodified DNA. On the other hand, DNMT1 works during DNA replication to copy the methylation pattern of the parental strand to the daughter strand [7]. Furthermore, this methylation can be reversed through the action of ten-eleven translocation (TET) oxidation of 5-methylcytosine (5mC) [8,9]. Histone methyltransferases (HMTs) module histone methylation at lysine residues on histone H3 and H4 [10]. The controlled balance of histone acetyltransferases (HATs) and histone deacetylates (HDACs) serve to regulate histone acetylation. Histone phosphorylation can also be used to modify DNA activity [10]. MicroRNAs can modulate gene expression by inhibiting mRNA translation or promotion its degradation [11].

**Figure 1:**
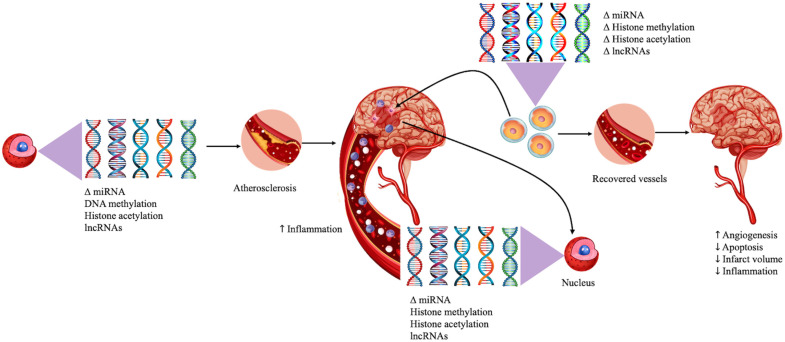
Epigenetic effects on atherosclerosis and neuroinflammation. This figure exemplifies the role of epigenetic modifications in promoting atherosclerosis, increasing the risk of stroke. It also illustrates how stroke can modify epigenetics worsening neuroinflammation. Stem cells have been shown to reverse these changes by increasing angiogenesis and decreasing inflammation, infarct volume, and apoptosis.

Many of these epigenetic modifications have been reported to modulate atherosclerosis development and progression, ultimately contributing to stroke pathogenesis [12]. Furthermore, epigenetics may play a major role in inflammatory responses following stroke [13] (Figure 1). While both genetics and epigenetics play a role in stroke risk factors, the potential for modification of epigenetic changes by lifestyle choices may also allow these alterations to serve as markers for disease progression [12]. The development of pharmaceuticals or cell-based therapies that target epigenetic aberrations may provide the potential to develop therapies that can promote plasticity to improve long-term recovery [14].

## 2. Epigenetic Changes Promoting Atherosclerosis

Hyperlipidemia, and by extension atherosclerosis, is a major risk factor associated with stroke [3]. Atherosclerosis is a complex disease with many different contributors and understanding the epigenetic processes behind it will help scientists and clinicians target it in preventative therapies to help at-risk patients avoid the debilitating impact of stroke. Factors such as high levels of low-density lipoproteins, triglycerides, and inflammation all play a role in determining an individual’s risk profile for developing atherosclerosis [15,16,17]. Recent research exploring epigenetic factors that play a role in the development of atherosclerosis may unveil new mechanisms and drug targets for future therapies. These can largely be broken down into DNA and RNA based mechanisms.

### 2.1. Histone Modifications

One promising therapeutic target for combating epigenetic regulated atherosclerosis in stroke involves the expression of histone deacetylases. For example, in models of histone deacetylase 3 (HDAC3) knockout mice, HDAC3 deficiency promotes the stabilization of atherosclerotic plaques by inducing fibrosis over existing lesion [18]. In humans, lower expression of HDAC3 correlates with higher expression of TGFβ1, which promotes fibrosis [18]. HDAC9 deficiency also induces fibrosis and increases the thickness of fibrotic caps over atherosclerotic lesions, while decreasing the presence of macrophages within the same lesions [19]. This is most likely due to the upregulation of the inflammatory NF-κB pathway by HDAC9, which when inhibited has a depressing effect on atherogenesis [20].

### 2.2. DNA Modifications

Aside from HDACs, another major player in epigenetics is the pattern of DNA methylation. Methylating certain genes can have significant downstream effects and provide potential therapeutic targets. For example, atherosclerotic arteries have much lower levels of methylated CpG islands in genes associated with angiogenesis, atherogenesis, and inflammation. These include the downregulation of FOXP1, upregulation of the AGE receptor ligand, and activators of NOTCH1 and EGFL7 [21]. On the other hand, the DNA methylation of genes such as the estrogen receptor beta gene (Erβ) also contributes to atherosclerosis by removing the cardioprotective effects of estrogen, including recovery from vascular injury [22,23]. Erβ knockout mice also displayed systolic and diastolic hypertension, which further supports Erβ’s multifaceted role in proper vascular function [24]. Interestingly, when blood flow is disturbed in the carotid artery of a mouse model, DNA methyltransferase (DNMT) activity increases and the inhibition of DNMT’s reduces levels of atherosclerotic lesion formation.

### 2.3. RNA Modifications

Common RNA mechanisms studied in epigenetics include the functions of miRNAs and other non-coding RNAs (ncRNAs). Changes in physiological conditions can precipitate alterations in ncRNA function and expression to create domino-like downstream effects. For example, miRNA-10a is downregulated in areas of the cardiovascular system with high levels of shear stress, such as the inner curvature of the aortic arch of rat models. This leads to higher levels of atherogenesis which can be rescued by the co-administration of retinoic acid receptor-α and retinoid X receptor-α agonists [25]. This is primarily through the inhibition of signaling pathways that promote the migration of inflammatory cells. Other miRNAs have the opposite effect of miRNA-10a; when upregulated by mechanical stress, they create a pro-atherogenic environment. For example, miRNA-712 is upregulated in vasculature disturbed flow and subsequently activates matrix metalloproteinases which stimulate an inflammatory and atherogenic state [26]. Still other miRNAs seem to demonstrate both pro- and anti-atherogenic responses, such as miR-155, where data shows that its deficiency can both decrease macrophage-induced inflammation and reduce levels of the anti-inflammatory cytokine IL-10 [27,28]. This discrepancy remains not fully understood, although it may have involved uncontrolled experimental variables that affected the outcome.

Aside from miRNAs, ncRNAs also play a role in epigenetic modification of atherogenic processes. For example, overexpression of long non-coding RNA (lncRNA) H19 is highly expressed in ApoE^−/−^ atherosclerotic mouse models, and its overexpression inhibits apoptosis in human umbilical vein endothelial cells [29]. This could point to a potential pro-inflammatory mechanism that increases risk of atherogenesis. Another example of a lncRNA closely associated with atherosclerosis is RNCR3, which is also significantly upregulated in mouse models [30]. Knocking out RNCR3 results in less proliferation and migration of endothelial and smooth muscle cells while simultaneously inducing apoptosis in these cells, resulting in an anti-atherogenic state [30].

Exploring epigenetic mechanisms behind atherosclerosis can be instrumental in stroke prevention. Atherogenesis is complex but the research behind its onset is ever-growing and provides promising therapeutic targets.

## 3. Epigenetic Changes in Stroke Promoting Inflammation

While ischemic injury in stroke is the primary cause of cell death, the secondary causes of cell death in stroke, such as excitotoxicity, oxidative stress, free radical accumulation, mitochondrial dysfunction, impaired neurogenesis, angiogenesis, vasculogenesis, and aberrant inflammation [31], are equally detrimental. A number of studies have demonstrated a role for epigenetics, including DNA methylation, histone acetylation, histone methylation, and miRNAs, in neuroinflammatory responses to ischemic injury, as previously reviewed [13]. Despite successful pre-clinical stroke therapy trials, the promising results do not transfer to the clinical setting. This disheartening patten may be due to complex and unique epigenetic patterns seen in every patient. Thus, epigenetics may offer groundbreaking therapies for diminishing neuroinflammation in stroke [13]. The following discussion focuses on histone modifications, ncRNAs, and miRNAs roles in stroke pathological inflammation due to their ample potential to be modified with pharmaceutical or cell-based therapies, as discussed later.

### 3.1. Histone Modifications

Histone methylation has been demonstrated in inflammatory responses to stroke. Histone lysine methylases (G9a lysine methylases) and demethylases (KDM4B lysine demethylases) are shown to regulate expression of TNF-α, a pro-inflammatory cytokine, and its effects on ICAM-1 and VCAM-1. Given these cellular adhesion molecules (CAMs) roles in neutrophil migration to the ischemic region, these epigenetic variations play a large role in inflammatory cell proliferation within the central nervous system (CNS) [32]. In mice, higher H3K9ac levels correspond to greater activation of microglia, thus perpetuating a pro-inflammatory environment [33]. In clinical settings, variations of histone acetylation (H3K9Ac) and methylation (H3K4me3) in stroke patients correlate with increased TNF-α concentrations as well. Lower TNF-α promotor methylation further correlates to increased stroke risk in patients. Given the role of methylation in reducing gene expression, it is logical that less methylation of the TNF-α promoter correlates with greater inflammation and stroke occurrence [34]. Altering pathological epigenetic modifications may be accomplished with histone deacetylases (HDAC) inhibitors. Two deacetylases, Sirt1 and Sirt3, are shown to control the blood–brain barrier integrity after stroke [35,36]. The impermeability of the BBB is vital to maintaining the CNS’s immune privilege and the stroke-induced leaky BBB allows for significant migration and damage from peripheral inflammatory cells [31]. In an in vitro stroke model of the BBB, comprising of human brain microvascular endothelial cells and astrocytes, Sirt1 suppression reduced BBB permeability. Contrarily, Sirt3 knockdowns further impaired the BBB integrity, suggesting that these two deacetylases play conflicting roles [35]. Intriguingly, Sirt3 is a mitochondrial form of the deacetylase, and, as noted, mitochondrial damage is a cause of secondary cell death after stroke [37]. Thus, the loss of mitochondria and this protective deacetylase may relate to cell death. Inhibiting HDACs in mice models also decreases TNF-α and NOS2, two inflammatory mediators, and increases the anti-inflammatory cytokine, IL-10, expression in microglia [33]. Furthermore, inhibiting HDACs leads to amplification of the anti-inflammatory and anti-oxidant Nrf2 pathway and its downstream products. The inhibition of HDACs protects in vitro neurons from oxygen-glucose deprivation models of ischemic stroke. These effects are also transferable to animals, reducing infarct volumes in mice models [38]. Nrf2 plays a well-established role in neuroprotection following stroke [39], and its amplification with HDAC inhibitors may be beneficial in clinical settings. With a better understanding of histone modifications and their role in stroke-induced neuroinflammation, pharmaceuticals or cell-based therapies may be developed to reduce this mechanism of secondary cell death following stroke.

### 3.2. DNA Modifications

Following stroke, DNA regions appear to be hypomethylated, but these epigenetic changes may fluctuate over time [13]. Methylation in certain regions may predict mortality following stroke, such as hypomethylation LINE-1 DNA [40]. LINE-1 DNA hypomethylation relates to levels of VCAM, an important protein involved in inflammatory cell migration. When hypomethylated, more VCAM can be expressed and inflammatory cell infiltration is facilitated. Contrarily, hypermethylation of thrombospondin-1 downregulates its anti-inflammatory and pro-angiogenesis effects [41,42]. A major player in these epigenetic responses and potential therapeutic target is DNA methyltransferases (DNMTs), which lead to immunosuppression in stroke [43], may be involved in cell death [44], and can even contribute to atherosclerosis as discussed above [45]. Another methylation modulator is methylenetetrahydrofolate reductase (MHTFR) [46], but its role in stroke risk and inflammation is debated.

### 3.3. RNA Modifications

ncRNAs, involved in regulating DNA and mRNA expression, have also been studied in the context of stroke neuroinflammation [47]. In a major epigenomic study on long lncRNA expression in cerebral microvascular endothelial cells after oxygen glucose deprivation, 217 lncRNAs were found to play a role in cellular changes in response to ischemia. Changes in lncRNA expression is observed in animal models as well. These genomic regions are preceded by numerous transcription factor binding sites, offering ample opportunities for therapeutic intervention [48]. Specific to inflammatory responses, an important lncRNA involved is transcribed by the gene *ANRIL,* located on chromosome 9p21.3. Increased *ANRIL* transcription directly relates to atherosclerosis severity. Thus, unsurprisingly, elevated ANRIL expression is also correlated to increased risk of stroke [49]. In 1657 cases of various stroke types (atherothrombosis, lacunar infarction, and hemorrhagic), the GG genotype of rs10757278 increased the risk of atherothrombotic stroke by 1.47× and hemorrhagic stroke by 1.6×. This genotype also increased the risk of stroke recurrence by 1.56× and mortality by 2.0× [50]. The detrimental impacts of ANRIL can be attributed to its role in enhancing angiogenesis via the VEGF pathway and increasing the pro-inflammatory IκB/NF-κB pathway [51]. The unique role of lncRNAs in inflammatory mediated stroke damage may offer a target for stroke treatments.

miRNAs play a major role in stroke neuroinflammation, and due to their propensity to target specific, small mRNA sequences to regulate translation, have promising treatment or biomarker potential (Table 1) [52]. MiR-223, for example, can downregulate inflammasome formation, and thus reduce cell death and inflammation, reduce edema, and ameliorate neurological dysfunction [53]. Another example is miR-126, a miRNA predominantly expressed in endothelial cells and downregulates VCAM expression. Thus, this miRNA plays a major role in reducing vascular inflammation and inflammatory cell migration to the region of stroke [54]. Similarly, miRNAs miR-98 and let-7g overexpression in mice reduce inflammatory cell migration past the BBB and amplify BBB integrity [55]. Let-7g miRNA is anti-inflammatory due to its ability to downregulate Toll-like receptors (TLR). TLR, in particular TLR-4, can respond to damaged proteins released by neuronal death after stroke and induce inflammation [56]. In vitro BBB models demonstrate that let-7i miRNA downregulates TLR4 expression following oxygen glucose deprivation. miR-181c also inhibits TLR4 expression and decreases NF-κB pathway activation to diminish inflammatory responses to hypoxia [57]. Furthermore, let-7i and mi-R155 can increase anti-inflammatory cytokines and growth factors (IL-4, IL-10, and BDNF) and decrease inflammatory mediators (IL-6 and iNOS) in microglia to promote recovery and anti-inflammation following stroke [58,59]. Interestingly, miR-155 can also have a pro-inflammatory role following stroke [58]. miR-155 plays a role in neuroinflammation and is upregulated by inflammatory cytokines, potentially contributing to BBB weakening [60,61,62,63]. Upregulating miR-155 increases monocyte and T-cell adhesion to the endothelium, allowing for facilitated infiltration into the infarct region [64]. Blocking miR-155 offers substantial therapeutic potential, and miR-155 inhibitors given to mice models show decreased inflammation, altered cytokine expression, and increased BDNF expression [62,63]. Given this complex and dichotomous impact on stroke inflammation, further research is warranted to determine the role of miR-155 in neuroinflammation. Let-7i, miR-98 and let-7g miRNA, however, may be beneficial if upregulated in cell-based stroke therapies.

miRNAs exhibit many of their anti-inflammatory properties via microglial modulation. Of major relevance in stroke pathology is miR-124 due to its primary expression in the CNS. In rats, this miRNA is elevated in the plasma following middle cerebral artery occlusion despite no changes in other miRNAs such as miR-122 and miR-133 [52]. This miRNA may also play a role in microglial suppression, making it a notable target for reducing microglial-induced neuroinflammation [65]. MiR-424 works similarly, as lentiviral overexpression of miR-424 in mice inhibits neuronal death and microglial activation [66]. MiR-let-7c-5p is yet another miRNA which can downregulate microglial activation, however, it is decreased in stroke patients’ plasma following ischemic injury. Overexpression in animals, however, reduces functional deficits and microglial activation, prompting future therapeutic consideration [67]. While prior studies have focused on ischemic stroke, overexpression of miR-132 in mouse models of hemorrhagic stroke similarly decreases activated microglia as well and decreases pro-inflammatory cytokines. miR-132 overexpression also restores BBB integrity and reduces cell death [51]. Additionally, in hemorrhagic stroke models, miR-367 inhibits inflammatory cytokine expression, reduces brain edema, and improves function. Once again, this is accomplished through modulating microglial function [68]. This miRNA also demonstrates anti-inflammatory properties by decreasing the NF-ĸB pathway [68]. In addition to decreasing microglial-induced inflammation, miRNAs may also prompt differentiation to the anti-inflammatory M2 microglial phenotype [13]. Inhibitors of miR-210 also decrease microglial activation and macrophage proliferation. These inhibitors further decrease pro-inflammatory cytokines (IL6, TNFα, IL1β, CCL2, and CCL3) and reduce ischemic injury when given to mice prior to and after MCAO. This inhibition can provide long-term functional improvement as well [69]. Modulation of miRNA function is a promising and plausible future for stroke therapeutics, as miRNAs can be very specifically targeted due to their propensity to bind specific mRNA sequences to exert their biological effects.

## 4. Stem Cells and Stroke

Stem cells have been largely studied as potential therapeutics for ischemic stroke due to their ability to differentiate into various cells of the nervous system. One of the primary objectives of acute stem cell therapy is to mitigate secondary injury following stroke, which is mediated by proinflammatory processes, mitochondrial dysfunction, oxidative damage, and apoptosis. Chronic treatment focuses on regeneration through the stimulation of processes such as vasculogenesis, neurogenesis, angiogenesis, and synaptogenesis [70]. Primary brain damage caused by infarction is irreversible and, accordingly, stem cell therapy for stroke aims to target these dysfunctional processes for treatment.

The properties of different sources of stem cells have been identified and studied in the context of stroke. One such class of cells is mesenchymal cells, which have been shown to exhibit neuroprotective and therapeutic properties. Different subtypes of mesenchymal cells show therapeutic potential for stroke. Induced pluripotent derived mesenchymal stem cells were found to exhibit neuroprotective effects on hypoxic PC12 cells through mitochondrial transfer [71].

A subset of mesenchymal cells called Muse cells are capable of specific differentiation into all three germ layers. Muse cell transplantation in experimentally induced stroke rats improves neurological and motor function with a high ratio of neuronal cell differentiation [72].

Embryonic stem cells demonstrate great potential as a therapeutic due to their ability to indefinitely divide and differentiate into cells from all three germ layers [70]. Intravenously delivered placental mesenchymal stem cells in rats with middle cerebral artery occlusion (MCAO) improved functional recovery and reduced lesion volume. These effects are potentially mediated by immunoreactions and astroglial cell activation [73]. Direct transplantation yields increased levels of neuron repair and angiogenesis with a smaller infarct [74]. In vitro and in vivo studies have shown contrasting increases in pro-inflammatory and anti-inflammatory mediators after transplantation. Though, the increased inflammation demonstrated through in vivo studies is also associated with the recruitment and mobilization of the stem cells, decreasing the effectiveness of concurrent immunosuppression. A limitation of embryonic stem cells is the controversy and ethics surrounding their use, which may limit their availability for research and treatment.

Bone marrow-derived mesenchymal stem cells (BM-MSCs) show promise as a therapeutic for stroke and potentially play a role in physiological neurorestoration. They are capable of differentiation into osteogenic, chondrogenic, and adipogenic cells [75]. During injury, bone marrow stem cells can traverse the bloodstream towards the site of injury and cross the blood–brain barrier in the process [76]. Intravenously administered human BM-MSCs showed somatosensory and cognitive benefits, increased vascular density, and increased angiogenic factors [77]. Similarly, transplantation of MSC-derived microvesicles utilizing BM-MSCs decreased inflammation and increased angiogenesis and neurogenesis in the rat model of ischemic stroke [78].

Another type of stem cell found in bone marrow is hematopoietic stem cells (HSCs), which can differentiate into all types of blood cells and are recruited by chemokines and other chemotactic factors [79]. Molecules such as G-CSF, can be used in stroke treatment to induce the recruitment and mobilization of HSCs [80]. Hematopoiesis following ischemia is myeloid-favored with formation of neutrophils and inflammatory monocytes with lower levels of lymphocyte precursors [81]. Use of HSCs is limited due to the potential for adverse effects, namely inflammation [82].

Yet, another class of stem cells which has shown therapeutic potential for ischemic stroke is neural stem cells (NSCs), which can differentiate into neurons and glial cells such as astrocytes and oligodendrocytes [83]. Sources commonly used for NSCs include various fetal tissue lines such as pluripotent stem cells. Implantation with NSCs aids with recovery after ischemic stroke and reduces the volume of the damaged area and associated inflammation [84,85]. Endogenous NSCs normally proliferate and migrate to the region of injury after hypoxia but are not sufficient for substantial neurogenesis [86,87]. One therapeutic mechanism of NSCs is the secretion of paracrine signals such as bFGF, VEGF, and EGF. A mitochondrial transfer mechanism is also possible, in which injury is reduced by restoring mitochondrial and aerobic function, which is also associated with neuronal differentiation [88]. Finally, NSCs can attenuate the immune system. Excessive inflammation and autophagy occurs and leads to cell death after ischemia, which can be inhibited by NSCs, promoting functional recovery as demonstrated by behavioral studies [89].

## 5. Enhancing the Stem Cell Epigenome for Treatment of Ischemic Stroke

In the setting of ischemic stroke models, many recent murine and cell culture studies involving administration of cell products (e.g., exosomes) from stem cells demonstrate promising neurological recuperation. Though it is historically understood that stem cells improve neurological status following cerebral ischemia, the following studies attempt to elicit the epigenetics involved and thus possible treatment modalities. A comprehensive list of the preclinical trials on epigenetic modification to stem cell therapy as a treatment modality for ischemic stroke is outlined in Table 2.

### 5.1. Bone Marrow-Derived MSCs

Multipotent BMSCs were epigenetically modified to overproduce miR-133b; exosomes from these cells were then harvested and administered intra-arterially to rats following middle cerebral artery occlusion (MCAO). These rats had improved neurological function and plasticity compared to rats injected with exosomes from BMSCs infected with blank vector, or phosphate-buffered saline. In an additional experiment, cultured astrocytes subjected to the oxygen and glucose deprivation (OGD) model were incubated with exosomes from naïve BMSCs, miR-133b downregulated BMSCs, or miR-133b-overexpressing BMSCs. Compared to naïve treatment, exosomes from miR-133b-overexpressing BMSCs significantly increase the number of exosomes released from the OGD astrocytes, whereas exosomes from miR-133b downregulated BMSCs decrease the number released. Exosomes harvested from the OGD astrocytes (cultured with exosomes from miR-133b-overexpressing BMSCs) also increase neurite branching in cultured cortical embryonic rat neurons [90].

Rats that underwent the MCAO model then intravenously received either miR-17-92 cluster-enriched exosomes (harvested from BMSCs infected with a miR-17-92 cluster plasmid), control BMSC exosomes, or liposomes. Compared to the control liposome group, both exosome-recipient groups demonstrated increased neurological function; however, the miR-17-92 cluster-enriched exosomes were significantly superior. Factors assessed include neurological function, oligodendrogenesis, neurogenesis, and neurite remodeling/dendrite plasticity within the ischemic site. miR-17-92 cluster-enriched exosomes inhibit phosphatase and tensin homolog, thus increasing the activation of the pathway involving PI3K/protein kinase B/mechanistic target of rapamycin/glycogen synthase kinase 3β [91].

MiR-126 acts upon the stromal cell-derived factor 1/C-X-C chemokine receptor type 7 signaling pathway. Mice demonstrate decreased infarction volume and increased angiogenesis following infusion with bone marrow-derived endothelial progenitor cells (EPC) treated with miR-126 [92].

In both rat MCAO and microglia OGD models, administration of exosomes from mesenchymal stem cells overexpressing miR-223-3p decreases infarct volume, and improves neurological deficits, learning, and memory. Within the ischemic zone, proinflammatory agents are suppressed and anti-inflammatory agents are promoted. Similar to the previous study, miR-223-3p promotes M2 microglial transformation [93].

Exosomes were collected from bone marrow-derived mesenchymal stem cells overexpressing miR-138-5p and cultured with astrocytes subjected to OGD. Under these conditions, astrocytes demonstrate increased proliferation, and decreased apoptosis and inflammation. In a separate experiment, the modified exosomes were administered to mice following MCAO, ameliorating neuronal injury [94]. The mechanism is via miR-138-5p’s inhibition of lipocalin 2, an iron transport protein promoting neuronal apoptosis secreted by astrocytes in the setting of neurodegeneration [95,96].

In the setting of MCAO, mice have decreased miR-542-3p and increased Toll-like receptor 4 (TLR4); of note, these molecules bind each other. Adeno-associated virus packaged miR-542-3p reduces infarction area, number of degenerating neurons, expression of inflammatory agents, and inflammatory cell infiltration. In a separate experiment, astrocytes undergoing OGD and exposed to miR-542-3p packaged in mesenchymal stem cells, show decreased apoptosis and reactive oxygen species. The mechanism involves the inhibition of TLR4 [97].

In the setting of MCAO, rats have decreased miR-150-5p and increased Toll-like receptor 5 (TLR5). Vesicles from bone marrow mesenchymal stem cells were isolated and administered to MCAO rat models, improving neurological function, and decreasing neuronal apoptosis and inflammatory factors. The mechanism involves inhibition of TLR5 [98].

In MCAO rat models, exosomes from bone marrow mesenchymal stem cells transfer miR-23a-3p, which polarizes microglia to their M2 form [99].

Extracellular vesicles from bone marrow stromal cells ameliorate neurological injury and blood–brain barrier permeability in MCAO mice and microglia/astrocyte OGD models. This phenomenon is mediated via miR-124 that may act on peroxiredoxin 1 [100].

Bone marrow mesenchymal stem cell-derived exosomes containing miR-455-3p ameliorate hippocampal neuronal injury in MCAO mice models and OGD cell models. The mechanism is via targeting of PDCD7 [101].

Mesenchymal stem cell-derived extracellular vesicles harboring miR-93 demonstrate improvement in MCAO rat models and OGD hippocampal neuron models. MiR-93 targets histone deacetylase 4 (HDAC4). HDAC4 deacetylates and thus inhibits the antiapoptotic molecule Bcl-2, leading to increased infarct volume and neuron apoptosis, and worsened neurobehavioral impairments [102].

Exosomes from bone marrow mesenchymal stem cells upregulate miR-21-5p and thus improve neurological function and reduce infarct volume in mouse stroke models. In vitro studies involving human umbilical vein endothelial cells demonstrated increased expression of VEGF, VEGFR2, Ang-1, and Tie-2 following exposure to exosomes [103].

### 5.2. Adipose-Derived MSCs

A separate study first upregulated miR-126 in adipose derived stem cells, then harvested and intravenously administered exosomes to control and stroke model rats. Following stroke, such rats exhibit improved neurological functional recovery, decreased neuronal death, and increased cell proliferation. These modified exosomes also inhibit microglial activation and expression of proinflammatory agents [104].

Rats demonstrate decreased area of cerebral infarction following infusion with adipose-derived stem cells treated with miR-30d-5p. The mechanism is via the inhibition of autophagy-mediated microglial polarization to M1 [105].

Contrary to the protective findings of miRs thus far, miR-21-3p is elevated in the setting of MCAO and reduces MAT2B expression in neural cells. The adipose-derived mesenchymal stem cell and its exosomes suppress miR-21-3p, thus protecting the blood–brain barrier, and inhibiting inflammation and apoptosis [106].

Neurons were exposed to OGD and then cultured with either adipose-derived mesenchymal stem cells (ADMSC) or ADMSC-secreted vesicles. Both therapies reduce cell death, and the mechanism is via decreased autophagy and p53-BNIP3 activity. Interestingly, ADMSC’s effects are lost when treated with an inhibitor of exosome secretion. Understanding that miR-25-3p is the most expressed miRNA in ADMSC-vesicles involved with the p53 pathway, the investigators discovered that an oligonucleotide mimic decreases cell death, whereas an anti-oligonucleotide increases autophagy and death. This phenomenon is observed similarly in mice models [107].

Vesicles from adipose-derived stem cells overexpressing miR-31 improve neurological function and reduce infarct volume and neuronal apoptosis in MCAO mice models. In neuron OGD models, the vesicle therapy decreases apoptotic factors caspase-3 and Bax and increased survival. The mechanism is via miR-31 binding to the 3′-untranslated region of TRAF6, thus inhibiting TRAF6 and subsequently inhibiting IRF5 (IRF5 promotes neuron apoptosis in the setting of OGD) [108].

ADMSCs and ADMSC-derived vesicles were cultured with neurons subjected to OGD. Consistent with the literature, this results in decreased neuronal apoptosis. Unique to this study, it was found that miR-22-3p is upregulated in ASC-vesicles; treatment with miR-22-3p inhibitor reverses the beneficial effects, increasing apoptosis. Mechanistically, miR-22-3p binds KDM6B, thus inhibiting its downstream effects on BMP2/BMF [109].

Similarly, in mice MCAO models, intravenous injection of vesicles from adipose-derived stem cells promotes M2 polarization. The latter study also demonstrates decreased brain atrophy volume, improved neuromotor and cognitive functions, attenuated loss of oligodendrocytes, increased angiogenesis, and proliferation of endothelial cells. MiRNA in these vesicles target STAT1 and PTEN, adjusting microglial polarization [110].

### 5.3. Umbilical MSCs

Exosomes from human umbilical cord mesenchymal stem cells expressing miR-146a-5p reduce microglia-mediated inflammation in OGD models, and both reduce infarct volume and ameliorate behavioral deficits and microglia activation in murine stroke models. The mechanism is via inhibition of the IRAK1/TRAF6 pathway [111].

### 5.4. Urine-Derived MSCs

When exosomes are harvested from urine-derived stem cells and administered intravenously to rat stroke models, the rats show enhanced neurogenesis and recovery. An additional experiment subjected neural stem cells to OGD along with the exosomes; the therapy promotes neuronal proliferation and differentiation. The mechanism may involve exosomal miR-26a’s inhibitory effect on histone deacetylase 6 [112].

**Table 2:** Epigenetic Stem Cell Studies for Stroke Therapy. This table outlines cell-based preclinical trials utilizing epigenetic modifications and interactions to enhance ischemic stroke recovery.

Citation	Sample	Cell Type	Route	Dosage	Results
Xin et al. (2017) [90]	MCAO rats	BMSC-derived exosomes	Intravenous	3 × 10^11^ particles	miR-133b overexpression induces astrocyte release of neurite-promoting exosomes for enhanced neural plasticity and restoration of neurological function.
Xin et al. (2017) [91]	MCAO rats	BMSC-derived exosomes	Intravenous	100 μg	miR-17-92 cluster-enriched exosomes improved neurological function and induced oligodendrogenesis, neurogenesis, and neuron plasticity.
Shan & Ma (2018) [92]	MCAO mice	Endothelial progenitor cells	Intravenous	2 × 10^5^ cells	Endothelial progenitor cells pretreated with miR-126 decreased infarct volume and increased angiogenesis by upregulation of the CXCR7 signaling pathway.
Jiang et al. (2018) [105]	MCAO rats	ADSC-derived exosomes	Intravenous	80 μg	Exosomes containing miR-30d-5p reduced infarct area, suppressed autophagy, and promoted M2 microglial/macrophage polarization.
Geng et al. (2019) [104]	MCAO rats	ADSC-derived exosomes	Intravenous	Not specified	miR-126 modification decreased neuron cell death, enhanced neuron proliferation, and inhibited microglia activation.
Deng et al. (2019) [94]	MCAO mice	BMSC-derived exosomes	Not specified	Not specified	miR-138-5p overexpression promotes astrocyte proliferation and downregulates inflammation through inhibition of lipocalin 2.
Li et al. (2019) [106]	MCAO rats	ADMSCs	Intravenous	6 × 10^6^ cells	Reduced elevated levels of miR-21-3p, inhibiting apoptosis, suppressing inflammation, and stabilizing the BBB.
Ling et al. (2020) [112]	MCAO rats	USC-derived exosomes	Intravenous	1 × 10^11^ particles	Promoted NSC proliferation and differentiation. Neurogenic effects attributed to inhibition of histone deacetylase 6.
Zhao et al. (2020) [93]	MCAO rats	BMSC-derived exosomes	Intravenous	200 μL	miR-223-3p overexpression decreased cerebral infarct volume and improved neurological recovery in learning/memory task via M2 microglia polarization.
Kuang et al. (2021) [107]	MCAO mice	ADMSC-derived EVs	Intravenous	2 × 10^6^ particles	miR-25-3p promoted neuroprotection through regulation of autophagy, reducing infarct size and neurological recovery.
Cai et al. (2021) [97]	MCAO mice	BMSC-derived exosomes	Intraventricular	2 × 10^10^ genome copies	miR-542-3p inhibited TLR4 to downregulate glial cell inflammation.
Lv, Li, & Che (2021) [108]	MCAO mice	ADSC-derived EVs	Intravenous	150 μg	miR-31 improved neurological function and reduced the expression of apoptotic factors such as cleaved caspase-3 and BAX by inhibiting IRF5 and TRAF6.
Zhang et al. (2021) [111]	MCAO mice	hUMSC-derived exosomes	Intravenous	50 μg	miR-146a-5p improved neural deficits following ischemic stroke by downregulating microglia-induced neuroinflammation via the IRAK1/TRAF6 pathway.
Zhang et al. (2021) [109]	MCAO rats	ADSC-derived EVs	Intraventricular	300 μg/kg	miR-22-3p ameliorated ischemia/reperfusion injury, decreased neuronal apoptosis, inhibited effects of KDM6B histone demethylase.
Li, Bi, & Yang (2022) [98]	MCAO rats	BMSC-derived exosomes	Intraventricular	100 μg/kg	miR-150-5p targeted TLR5 resulting in improved neurological function, reduced inflammation, and inhibition of neuronal apoptosis.
Dong et al. (2022) [99]	MCAO rats	BMSC-derived exosomes	Intravenous	3 × 10^11^ particles	Transference of miR-23a-3p induces M2 polarization and deactivation of microglia, improving injury in cerebral infarction.
Hu et al. (2022) [110]	MCAO mice	ADSC-derived EVs	Intravenous	700 μg	Upregulated miRNAs promoted expression of STAT1 and PTEN. Administration reduced brain atrophy, improved neurological function, induced angiogenesis, and promoted M2 microglial polarization.
Tian et al. (2022) [100]	MCAO mice	BMSC-derived EVs	Intravenous	20 μg/mL	Expression of miR-124 reduced volume of infarct damage by inhibiting microglial activation and downregulating BBB permeability.
Gan & Ouyang (2022) [101]	MCAO mice	BMSC-derived exosomes	Intraventricular	100 μg/kg	Administration of BMSC-Exos upregulated miR-455-3p, leading to an inhibition of programmed cell death 7 gene. Promoted neuroprotection by reducing hippocampal neuron apoptosis.
Shi et al. (2022) [102]	MCAO rats	BMSC-derived EVs	Intraventricular	300 μg/kg	MiR-93 suppressed histone deacetylase 4, reducing apoptosis of hippocampal neurons and hypoxic-ischemic brain damage.
Hu et al. (2022) [103]	MCAO mice	BMSC-derived exosomes	Intravenous	25 μg or 50 μg	Administration of BMSC-Exos upregulated miR-21-5p, leading to a promotion of angiogenesis.

## 6. Conclusions

The role of epigenetic modifications in stroke pathophysiology has resulted in a plethora of intriguing and groundbreaking new research. The specificity of some of these epigenetic changes, such as with miRNAs and ncRNAs, allow for targeted therapeutic intervention. With a better understanding of how these epigenetic modifications influence inflammation and atherosclerosis, cell-based therapies can be developed to directly modulate these changes and mitigate stroke risk and damage.

## Figures and Tables

**Table 1:** miRNAs involved in stroke inflammatory responses. This table describes the various roles of microRNA in modulating inflammatory responses after stroke.

miRNA	Model	Results	Citations
miR-223	Mouse model of hemorrhagic stroke	Downregulates inflammasome formation, and thus reduce cell death and inflammation, reduce edema, and ameliorate neurological dysfunction	[53]
miR-126	Low-density lipoprotein-injured vascular endothelial cells from rat thoracic aorta	Downregulates VCAM expression to reduce vascular inflammation and inflammatory cell migration to the region of stroke	[54]
miR-98	In vitro: Brain microvascular endothelial cells In vivo: Mice	Reduces inflammatory cell migration past the BBB and amplify BBB integrity	[55]
let-7g	In vitro: Brain microvascular endothelial cells In vivo: Mice In vitro: Human embryonic kidney (HEK) 293 cells	Reduces inflammatory cell migration past the BBB and amplify BBB integrity Downregulates TLR-4 which can respond to damaged proteins released by neuronal death after stroke and induces inflammation	[55,56]
let-7i	Human brain microvascular endothelial cells (HBMECs) in an oxygen-glucose deprivation (OGD) model	Downregulates TLR4 expression following oxygen glucose deprivation Can increase anti-inflammatory cytokines and growth factors (IL-4, IL-10, and BDNF) and decrease inflammatory mediators (IL-6 and iNOS) in microglia to promote recovery and anti-inflammation following stroke	[58,59]
miR-181c	BV-2 microglial cell line and primary cultured rat microglial cells	Inhibits TLR4 expression and decreases NF-κB pathway activation to diminish inflammatory responses to hypoxia	[57]
mi-R155	Human brain microvascular endothelial cells (HBMECs) in an oxygen-glucose deprivation (OGD) model Oxygen-glucose deprivation/reoxygenation (OGD/R)-treated N2a cells MCAO mouse model	Can increase anti-inflammatory cytokines and growth factors (IL-4, IL-10, and BDNF) and decrease inflammatory mediators (IL-6 and iNOS) in microglia to promote recovery and anti-inflammation following stroke Upregulated by inflammatory cytokines, potentially contributing to BBB weakening. miR-155 inhibitors given to mice models show decreased inflammation, altered cytokine expression, and increased BDNF expression	[60,61,62,63]
miR-124	Rat surgical stroke model Neuroinflammatory mice model	Elevated in the plasma following middle cerebral artery May also play a role in microglial suppression	[52,65]
miR-424	Ischemic stroke mouse model	Lentiviral overexpression inhibits neuronal death and microglial activation	[66]
miR-let-7c-5p	Plasma of patients with ischemic stroke and MCAO mice model	Can downregulate microglial activation, however, it is decreased in stroke patients’ plasma following ischemic injury Overexpression in animals reduces functional deficits and microglial activation, prompting future therapeutic consideration	[67]
miR-132	Mouse model of hemorrhagic stroke	Decreases activated microglia as well and decreases pro-inflammatory cytokines in hemorrhagic stroke Overexpression also restores BBB integrity and reduces cell death	[51]
miR-367	In vitro microglia and mouse model of hemorrhagic stroke	Inhibits inflammatory cytokine expression, reduces brain edema, and improves function in hemorrhagic stroke Anti-inflammatory properties by decreasing the NF-ĸB pathway	[68]
miR-210	MCAO model of mice	Inhibition decreases microglial activation and macrophage proliferation, decrease pro-inflammatory cytokines, and reduce ischemic injury. This inhibition can provide long-term functional improvement as well	[69]
